# Excess Adiposity Without Obesity in a High-Risk Population

**DOI:** 10.1001/jamanetworkopen.2025.35194

**Published:** 2025-10-03

**Authors:** Alexandra B. Palmer, Rashedeh Roshani, Joseph B. McCormick, Susan P. Fisher-Hoch, Jennifer E. Below, Kari E. North, Penny Gordon-Larsen

**Affiliations:** 1 Department of Epidemiology, Gillings School of Global Public Health, The University of North Carolina at Chapel Hill; 2 Department of Medicine, Division of Genetic Medicine, Vanderbilt University School of Medicine, Nashville, Tennessee; 3 Vanderbilt Genetics Institute, Vanderbilt University Medical Center, Nashville, Tennessee; 4 Department of Epidemiology, The University of Texas Health Science Center at Houston School of Public Health, Brownsville Regional Campus, Brownsville; 5 Department of Medicine, Division of Cardiovascular Medicine, Vanderbilt University Medical Center, Nashville, Tennessee; 6 Department of Epidemiology, UTHealth Houston School of Public Health, Houston, Texas; 7 Department of Internal Medicine, McGovern Medical School, UTHealth Houston, Texas; 8 Department of Nutrition, Gillings School of Global Public Health, University of North Carolina at Chapel Hill

## Abstract

This cross-sectional study estimates excess adiposity in Mexican-American individuals with and without obesity enrolled in the Cameron County Hispanic Cohort.

## Introduction

Body mass index (BMI; calculated as weight in kilograms divided by height in meters squared) remains widely used to define obesity, yet it has long been criticized for its limited ability to distinguish individuals with and without excess body fat.^1^ This imprecision can result in underdiagnosis of excess adiposity among individuals at high risk and overdiagnosis of those without excess adiposity. Recognizing these shortcomings, a recent *Lancet* Commission called for a new obesity diagnostic framework, with initial screening by BMI and subsequent confirmation of excess adiposity.^2^

Aryee et al^3^ reported the prevalence of obesity defined by BMI alone to be nearly identical to that of excess adiposity, measured via waist circumference (WC), dual-energy x-ray absorptiometry (DEXA)-derived total body fat percentage (TBF), or BMI of 40 or more, in National Health and Nutrition Examination Study (NHANES). Our objective was to challenge the understanding of these findings^3^ in a high-risk population of self-reported Mexican-American individuals living at the US and Mexico border with high prevalent obesity exceeding that of NHANES.

## Methods

The Cameron County Hispanic Cohort (CCHC) is a population-based cohort study recruited from randomly selected households in Cameron County, Texas, under informed consent from 2014 to 2023.^4^ We included 1465 CCHC participants aged 18 years or older with DEXA and anthropometry, including weight, height, and WC. This study was deemed exempt by the University of North Carolina Institutional Review Board. We estimated obesity using BMI of 30 or more and excess adiposity using elevated WC (men, 102 cm or more; women, 88 cm or more), elevated TBF percentage (men, 25% or more; women, 35% or more), or a BMI of 40 or more, per *Lancet* commission recommendations,^2^ among participants with BMI of less than 25, 25 to 30, and 30 or more. Data were analyzed using R version 4.1.0 (R Project for Statistical Computing). This cross-sectional study followed the STROBE reporting guideline.

## Results

Among 1465 CCHC adults (mean [SD] age, 51.8 [15.2] years; 984 females [67.2%]), 756 participants (51.6%) had obesity according to BMI (Figure), and 1378 participants (94.1%) had excess adiposity overall: 1088 had elevated WC [74.3%], 1317 had elevated TBF [89.9%], and 109 had a BMI of 40 or more [7.4%] with variation by age and sex (Table). Of individuals with a BMI of 30 or more, almost all (755 [99.9%]) had confirmed adiposity by at least 1 criterion: 731 had elevated WC (96.7%), 726 had elevated TBF (96.0%), and 109 had a BMI of 40 or more (14.4%). Excluding DEXA and using only anthropometric indicators (WC or BMI of 40 or more), 732 individuals (96.8%) with obesity by BMI met criteria for excess adiposity. Additionally, 623 participants (87.9%) with a BMI of less than 30 also had excess adiposity, underscoring limitations of BMI alone in detecting true adiposity.

**Figure.**
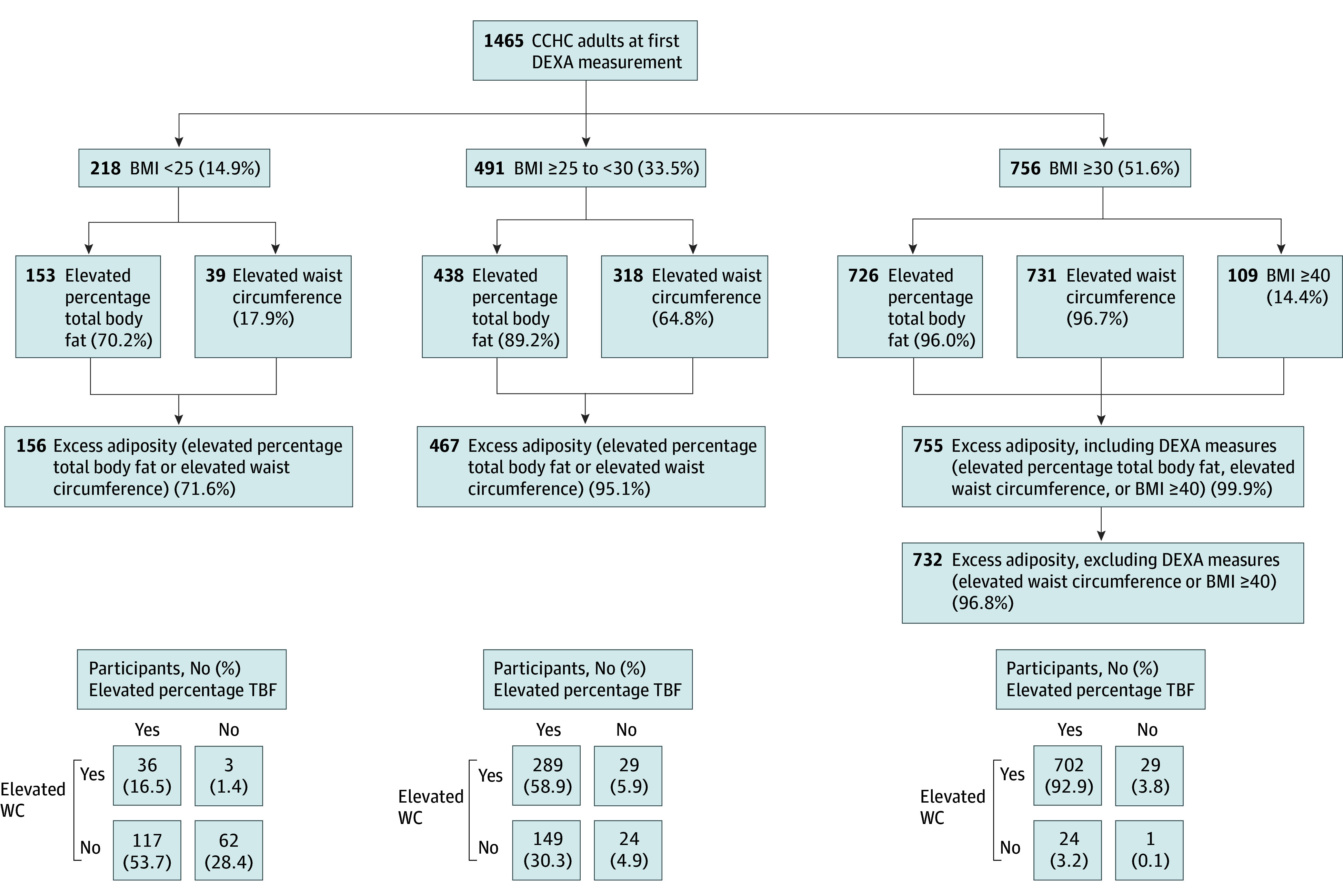
Prevalence of Excess Adiposity^a^ Among Participants With and Without Overweight or Obesity^b^ Classified Using BMI in the Cameron County Hispanic Cohort (CCHC) BMI, body mass index (calculated as weight in kilograms divided by height in meters squared); DEXA, dual-energy x-ray absorptiometry; TBF, total body fat; WC, waist circumference. ^a^Excess adiposity defined as elevated WC (≥102 cm for men; ≥88 cm for women), or elevated percentage TBF (≥25% for men; ≥35% for women), or a BMI of 40 or more. ^b^Overweight: BMI ≥25 to <30; obesity: BMI ≥30.

**Table.** Prevalence of Excess Adiposity Among Adult Participants in the CCHC Study, Overall and by BMI, Sex, and Age

Characteristics	Elevated % TBF^a^	Elevated WC^b^	BMI ≥40	Elevated % TBF or WC or BMI ≥40	Elevated WC or BMI ≥40
**Overall (n = 1465)**					
Total^c^	1317 (89.9)	1088 (74.3)	109 (7.4)	1378 (94.1)	1089 (74.3)
Sex					
Male	413 (85.9)	275 (57.2)	20 (4.1)	432 (89.8)	275 (57.2)
Female	904 (91.9)	813 (82.6)	89 (9.0)	946 (96.1)	814 (82.7)
Age group, y					
18-34	194 (82.2)	132 (55.9)	21 (8.9)	202 (85.6)	132 (55.9)
35-54	506 (92.0)	411 (74.7)	46 (8.4)	527 (95.8)	411 (74.7)
≥55	617 (90.9)	545 (80.3)	42 (6.2)	649 (95.6)	546 (80.4)
**Participants below overweight threshold (BMI <25; n = 218)**					
Total^c^	153 (70.2)	39 (17.9)	NA	156 (71.6)	39 (17.9)
Sex					
Male	42 (60.9)	3 (4.3)	NA	42 (60.9)	3 (4.3)
Female	111 (74.5)	36 (24.2)	NA	114 (76.5)	36 (24.2)
Age group, y					
18-34	37 (53.6)	5 (7.2)	NA	39 (56.5)	5 (7.2)
35-54	48 (81.4)	9 (15.3)	NA	48 (81.4)	9 (15.3)
≥55	68 (75.6)	25 (27.8)	NA	69 (76.7)	25 (27.8)
**Participants with overweight (BMI ≥25 to <30; n = 491)**					
Total^c^	438 (89.2)	318 (64.8)	NA	467 (95.1)	318 (64.8)
Sex					
Male	152 (84.0)	58 (32.0)	NA	160 (88.4)	58 (32.0)
Female	286 (92.3)	260 (83.9)	NA	307 (99.0)	260 (83.9)
Age group, y					
18-34	57 (87.7)	29 (44.6)	NA	61 (93.8)	29 (44.6)
35-54	163 (88.1)	109 (58.9)	NA	174 (94.1)	109 (58.9)
≥55	218 (90.5)	180 (74.7)	NA	232 (96.3)	180 (74.7)
**Participants with obesity (BMI ≥ 30; n = 756)**					
Total^c^	726 (96.0)	731 (96.7)	109 (14.4)	755 (99.9)	732 (96.8)
Sex					
Male	219 (94.8)	214 (92.6)	20 (8.7)	230 (99.6)	214 (92.6)
Female	507 (96.6)	517 (98.5)	89 (17.0)	525 (100.0)	518 (98.7)
Age group, y					
18-34	100 (98.0)	98 (96.1)	21 (20.6)	102 (100.0)	98 (96.1)
35-54	295 (96.4)	293 (95.8)	46 (15.0)	305 (99.7)	293 (95.8)
≥55	331 (95.1)	340 (97.7)	42 (12.1)	348 (100.0)	341 (98.0)

Abbreviations: BMI, body mass index (calculated as weight in kilograms divided by height in meters squared); CCHC, Cameron County Hispanic Cohort; TBF, total body fat; WC, waist circumference.

^a^ Elevated percentage TBF defined as 25% or more for men and 35% for women, measured via dual-energy x-ray absorptiometry.

^b^ Elevated WC defined as 102 cm or more for men and 88 cm or more for women.

^c^ Percentages are row percentages.

##  Discussion

In this study, we observed an alarmingly high prevalence of excess adiposity (94.1%) in this high-risk population, particularly among participants with overweight (BMI ≥25 to <30). We demonstrated considerable underdiagnosis of excess adiposity among individuals below the BMI criterion for obesity, none of whom would be captured in standard practice, despite their increased risk for adiposity-related diseases.

Most participants with a BMI of 30 or more had confirmed excess adiposity. Our finding is similar to Aryee et al,^3^ which found 98.4% confirmed adiposity among individuals with obesity via BMI in NHANES. Thus, we observed even higher confirmed adiposity in a population with a higher prevalence of obesity (51.6% in CCHC vs 39.7% in NHANES). These findings highlight that while BMI rarely misclassifies excess adiposity in individuals with a BMI or 30 or more, it may miss many individuals with excess adiposity in lower BMI ranges in this population.

Given DEXA’s impracticality for clinical or population screening—due to cost and specialized equipment—it is notable that anthropometry (WC) sufficiently confirmed excess adiposity in individuals with a BMI of 30 or more (99.9% confirmed via DEXA vs 96.8% using anthropometrics alone). However, DEXA was important for distinguishing excess adiposity in individuals with overweight.

Limitations include a relatively small sample size. The high prevalence of excess adiposity among individuals with a BMI of less than 30 is concerning, as they may be missed in routine screening and could benefit from earlier intervention to reduce the risk of adiposity-related adverse outcomes, as seen in individuals with a BMI of less than 30 who have excess adiposity.^5,6^

## Notes

Supplement.
Data Sharing Statement
