# Supplementary material for: Excess Adiposity Without Obesity in a High-Risk Population
Source: JAMA Netw Open. 2025 Oct 3;8(10):e2535194. doi: 10.1001/jamanetworkopen.2025.35194 (PMC12495492; doi:10.1001/jamanetworkopen.2025.35194)
Supplement: Supplement. — Data Sharing Statement [file jamanetwopen-e2535194-s001.pdf]

Data Sharing Statement

Data
Data available: Yes
Data types: Deidentified participant data
How to access data: https://sph.uth.edu/research/centers/hispanic-health/#TID-8d39067e-3bc7-4ebe-8734-4ed463f94bf1-2
When available: With publication

Supporting Documents
Document types: Statistical/analytic code
How to access documents: Palmer, Alexandra Bukowski <alexandra_bukowski@med.unc.edu>
When available: With publication

Additional Information
Who can access the data: With data use agreement
Types of analyses: Specific requests and collaboration agreements
Mechanisms of data availability: Signed data access agreement
